# Family Reading

**Published:** 1873-08

**Authors:** 


### FAMILY READING.

The character, extent of culture and elevation of individuals and families is indexed by the newspapers, magazines and books which they habitually read; this reading confirms the character, makes it more pronounced, elevates or degrades. A Christian prefers religious books, and becomes a more decided Christian. A business man prefers a newspaper which most abounds in business information as to ships, merchandise, and stocks and bonds. The trifling mind busies itself in reading trashy novels and dishwater love stories. Every reflecting person will naturally select that kind of reading for himself and those under him which will cherish and cultivate the traits and dispositions which are most desired. Every family, then, ought to select its reading for the year with reflection and judgment; the father and mother are responsible for the household, the children and the servants under them.

Every religious family owes it to itself and its Church to take the religious paper which is nearest the authorized organ of the society to which it belongs; and a wrong is done to that Church if its paper is not patronized in preference to any other religious newspaper, however superior it is, for patronage will help to make it superior. There is no liberality, in the true sense of the term, for a man to take a paper of another denomination while he neglects the paper of his own Church, any more than it is true liberality and a wise policy to go and hear strange preachers, and attend other churches than your own, even if of the same denomination. He is the most liberal church member who oftenest goes to his own meeting and most sustains his own minister; these are the only church members who are really of any account in building up the Church anywhere, and making it an aggressive and a progressive organization.

If, on the other hand, a man wants his sons and daughters to grow up thoughtful, inquiring, intelligent, and well informed as to the duties of life, instead of bringing into the family a promiscuous assortment of fictitious reading, with illustrations of all that is violent and vile, he will sedulously and peremptorily exclude these from his centre table and patronize magazines of acknowledged merit, known to give substantial reading.

The farmer who does not take some agricultural publication will lose more in any one year than would pay the subscription price of a dozen, many times multiplied, even if he cultivates but a score or two acres of land.

There are a few papers which belong to the farm which give instruction, and receive information on a great variety of subjects. The names of a number of these will be found in previous numbers of the JOURNAL, with the places and prices of publication.

There is one newspaper, the only one of the kind in the United States, which may be safely and profitably taken in every family, *The New York Daily Witness.* It costs but four dollars a year. The weekly contains the reading matter of the daily, and costs but one dollar a year. The daily gives the most important items of news, markets, stocks, and other valuable business information; the "opinions" of the press of all parties, with a considerable portion of reading matter, adapted to the capacities and needs of every member of the family. Besides the various political, mercantile and religious news, each paper contains miscellaneous reading matter suitable for all. The last issue has the headings of "The Three Last Calls," "Muffins for Tea," "Ferri Violents," "Little Peter," "Runaway Son," "Plowing," "Daily Bread," "Soul's Inquiries," "Comfort for Believers," "Crime and its Poverty," "Alms Houses," "Value of Sunday Schools to the Teachers," "Sins and Follies of the Working Classes," "Education in Humanity," "Italian Mission," "Fulton street Prayer Meeting Report." Surely it must be better to have such a paper go into city families and farmers' families—better for the children, better for the servants—than any ordinary daily paper, filled, as usual, with details of crime, indecencies, and wrong doing of every description, to say nothing of the irreligious and infidel tendencies of three fourths of all the "leading" newspapers of the country.

To take such a paper is not the whole of the duty of a Christian business man; he should give it his countenance by a liberal advertising patronage, especially as such a paper is almost exclusively taken by moral, and Christian, and educated readers—and, being such, are reliable as purchasers of what is advertised, and are more likely to appreciate what is valuable and useful, and more swift in their appreciation. Intelligent parents, teachers and employers are earnestly solicited to give this subject their thoughtful consideration, and thus promote moral and physical health everywhere, for everywhere and always they go together.

